# Magnetic spatiotemporal control of SOS1 coupled nanoparticles for guided neurite growth in dopaminergic single cells

**DOI:** 10.1038/s41598-020-80253-w

**Published:** 2020-12-31

**Authors:** Fabian Raudzus, Hendrik Schöneborn, Sebastian Neumann, Emilie Secret, Aude Michel, Jérome Fresnais, Oliver Brylski, Christine Ménager, Jean-Michel Siaugue, Rolf Heumann

**Affiliations:** 1 Department of Biochemistry II, Molecular Neurobiochemistry, Faculty of Chemistry and Biochemistry, Ruhr-Universität Bochum, 44801 Bochum, Germany; 2 Sorbonne Université, CNRS, Physico-Chimie des Électrolytes et Nanosystèmes Interfaciaux, PHENIX, 75005 Paris, France; 3 Technische Universität Braunschweig, Institut für Physikalische und Theoretische Physik, Biophotonik, Rebenring 56, 38106 Braunschweig, Germany; 4 Present Address: Department of Clinical Application, Center for iPS Cell Research and Application (CiRA), Kyoto University, Kyoto, 606-8507 Japan

**Keywords:** Biochemistry, Cell biology, Neuroscience, Stem cells, Diseases, Molecular medicine, Nanoscience and technology

## Abstract

The axon regeneration of neurons in the brain can be enhanced by activating intracellular signaling pathways such as those triggered by the membrane-anchored Rat sarcoma (RAS) proto-oncogene. Here we demonstrate the induction of neurite growth by expressing tagged permanently active Harvey-RAS protein or the RAS-activating catalytic domain of the guanine nucleotide exchange factor (SOS1cat), in secondary dopaminergic cells. Due to the tag, the expressed fusion protein is captured by functionalized magnetic nanoparticles in the cytoplasm of the cell. We use magnetic tips for remote translocation of the SOS1cat-loaded magnetic nanoparticles from the cytoplasm towards the inner face of the plasma membrane where the endogenous Harvey-RAS protein is located. Furthermore, we show the magnetic transport of SOS1cat-bound nanoparticles from the cytoplasm into the neurite until they accumulate at its tip on a time scale of minutes. In order to scale-up from single cells, we show the cytoplasmic delivery of the magnetic nanoparticles into large numbers of cells without changing the cellular response to nerve growth factor. These results will serve as an initial step to develop tools for refining cell replacement therapies based on grafted human induced dopaminergic neurons loaded with functionalized magnetic nanoparticles in Parkinson model systems.

## Introduction

In contrast to the peripheral nervous system (PNS), the regeneration of neurons in a diseased central nervous system (CNS) is limited, resulting in severe permanent disabilities^1^. If a neuron of the PNS loses its axon, the initiation and growth of a new axon are supported by its permissive Schwann cell environment, which facilitates an intrinsic regenerative response^2–4^. However, during embryogenesis, neurons of the CNS lose their ability to regenerate, partly because the environment of the adult brain is inhibitory for axonal growth and intrinsic regenerative responses are suppressed^3,5–7^.

In spite of this, several intracellular signaling molecules have been shown to counteract the inhibitory environment in adult CNS neurons^8,9^. One promising intracellular candidate for promoting neurite growth is the small GTPase H-RAS, which mimics neurotrophin signaling^10,11^, resulting in an enhanced regeneration response after injury^12^. Accordingly, fiber outgrowth is prevented by inhibiting H-RAS signaling after the introduction of anti-RAS antibodies or anti-RAS antigen-binding fragments (Fab) into the neuronal cytoplasm^13^. Furthermore, the transgenic overexpression of constitutively active H-RAS^V12^ in mouse facial motor neurons has neuroprotective effects and enhances sprouting and axonal regeneration^12,14^. In addition, activation of the RAS downstream effector B-rapidly accelerated fibrosarcoma (B-RAF) in retinal ganglia cells (RGCs) promotes axon growth after optic nerve crush^15^.

H-RAS belongs to the RAS family of small GTPases and is located at the inner plasma membrane due to anchoring by farnesylation and palmitoylation. Guanine exchange factors (GEFs) and GTPase-activating proteins (GAPs) regulate H-RAS by cycling the GTPase between guanosine triphosphate (GTP)-loaded “on” and guanosine diphosphate (GDP)-loaded “off” states. Upon the binding of growth factors to the corresponding receptor tyrosine kinases (RTKs), the GEF son of sevenless 1 (SOS1) is activated by phosphorylation and promotes the exchange of GDP to GTP, thereby converting inactive RAS-GDP to active RAS-GTP. Subsequently, active H-RAS localized at the cytoplasmic membrane recruits RAF by interacting with its RAS binding domain (RBD), which leads to the phosphorylation and dimerization of RAF. These events stimulate the kinase activity of RAF, resulting in propagation of the signal to MAPK/ERK kinase (MEK) and Extracellular signal-regulated kinase (ERK), inducing the translocation of ERK from the cytoplasm to the nucleus. In the nucleus, ERK modulates transcription factor activity that affects cell survival, cell motility, cell division, and neurite outgrowth^16,17^.

Neurons are polarized cells with highly specialized compartmentalization and specific signaling mechanisms^18^. During the formation of axons in primary neurons, H-RAS is enriched in the growth cone. Upon the inhibition of H-RAS, polarization is reduced, suggesting a corresponding role of endogenous H-RAS in the axonal tip^19^. In order to achieve directed growth, H-RAS or its effectors must be redistributed in a spatially controlled way to focus signaling at the defined submembrane regions and specifically in the tip of the fiber or the axonal growth cone. Unfortunately, the global overexpression of proteins in the RAS/RAF/MEK/ERK pathway does not allow for the spatial control of neuronal fiber growth.

The precise spatiotemporal control of cellular functions by the remote control of magnetic nanoparticles using static or oscillating magnetic fields is gaining attention in regenerative medicine^20^. Magnetic fields can non-invasively penetrate into deep tissue, enabling magneto-mechanical and magneto-molecular approaches to modulate cell behavior^21^.

Magneto-mechanical stimulation describes the translation of a magnetic field gradient to a mechanical force. The magnetic control of neurite growth has been described recently by using magnetic nanoparticles (MNPs) that were taken up into the cell through endocytic vesicles and therefore were present in enclosed membrane-confined compartments within the cellular cytoplasm^22–24^. Upon the application of an external magnetic field, the membrane-confined MNPs were attracted and thereby generated a mechanical stimulus within the cell. Due to this stimulus, stretch-growth and a change in direction of the neurite growth were achieved^22–24^. Furthermore, cell migration and neurite outgrowth can be directed by the forces produced by a switchable parallelized array of micro-magnetic pillars, following the passive uptake of nanoparticles^25^. However, whether MNP-loaded endosomes can promote extensive directional elongation of the axon, which is needed to bridge long distances between pathologically disconnected brain regions such as in Parkinson`s disease, remains elusive.

In contrast to the above-mentioned approaches, we here describe a complementary magneto-molecular signaling approach based on protein-functionalized nanoparticles that are applied directly to the cytoplasm and are not taken up into endosomes. We generated respective HaloTag (HT) fusion proteins of constitutively active H-RAS^V12^ and the catalytic domain of its GEF, SOS1 (SOS1cat), and demonstrated their ability to induce fiber outgrowth. After cytoplasmic capturing of the fusion proteins by HT ligand (HTL)-functionalized, γ-Fe_2_O_3_@SiO_2_ core–shell magnetic nanoparticles (HTL-MNPs), we show that magnetic guidance of the HT fusion protein-bound MNPs from the cytoplasm to the neurite tip, where endogenous H-RAS protein is accumulated and the growth cone explores its direction of elongation. In order to scale up the cytoplasmic loading of MNPs, we performed the “scrape-loading” technique and tested the response of PC12 cells to morphological differentiation by nerve growth factor (NGF).

## Results

### Experimental design and generation of HT fusion proteins

**Figure 1:**
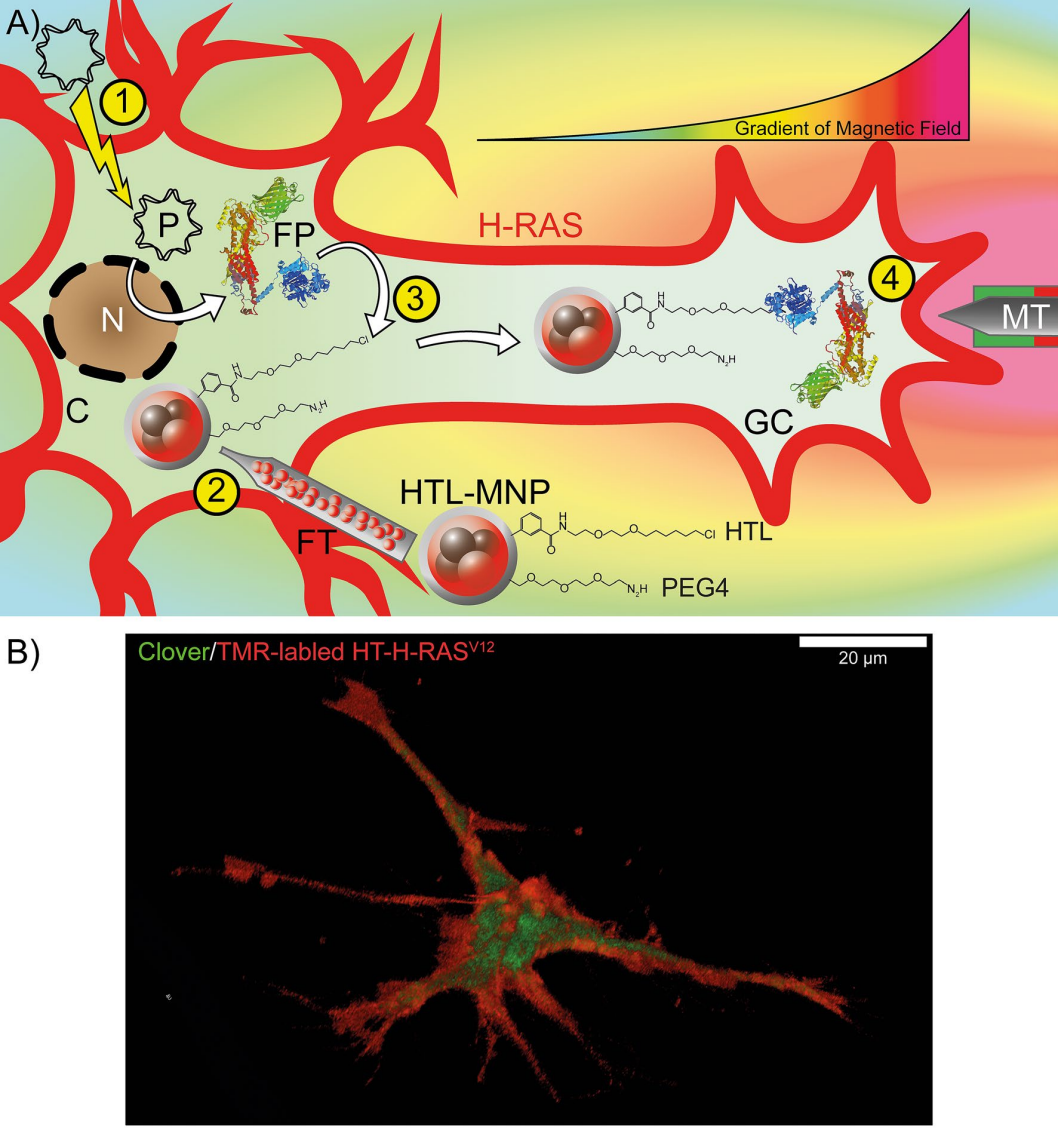
(**A**) Schematic outline of the experimental design. (1) The plasmid coding for HT-SOS1cat-Clover is electroporated, and the protein is expressed in the cell. (2) 48 h after the electroporation, magnetic nanoparticles functionalized with HaloTag Ligand (HTL-MNPs) are microinjected into the cell. (3) HT-SOS1cat-Clover is captured by the HTL-MNPs in the cytoplasm due to specific covalent binding of the HT to its ligand. (4) A magnetic tip is moved towards the neurite extension, which causes an accumulation of HT-SOS1cat-coupled MNPs in the growth cone in response to the applied magnetic field. Thus, HT-SOS1cat-Clover is translocated into the vicinity of the endogenous, membrane-anchored H-RAS, which cycles between its inactive GDP-bound and signaling active GTP-bound conformations. The magnetic field gradient is indicated by the curve above the growth cone (high magnetic field in red, low magnetic field in green; arbitrary units) and by the corresponding color gradient of the background. *C* cytoplasm, *FP* HT-SOS1cat-Clover fusion protein, *FT* Femtotip, *GC* growth cone, *MT* magnetic tip, *N* nucleus, *P* plasmid. Membrane-anchored H-RAS is indicated by the broad and thick red outline of the cell. (**B**) A three-dimensional confocal image of a PC12 cell transfected with HT-H-RAS^V12^-IRES-Clover after in-cell binding of the red-fluorescent cell-permeable dye TMR-HTL 2 days after the transfection. Note that morphological differentiation was achieved in the absence of NGF due to the neurite promoting activity of H-RAS^V12^ after the transfection (see Fig. 3). Three-dimensional reconstruction was performed using images from 55 Z-layers that cover a total distance of 18.9 µm. The scale bar represents 20 µm.

In order to direct the fiber growth, focal H-RAS activation is required. The schematic outline in Fig. 1A shows the electroporation of the plasmid coding for HT-SOS1cat-Clover and its expression (1), the injection of HTL-MNPs (2), the cytoplasmic capturing of SOS1cat fusion protein by HTL-MNPs (3), and the magnetic translocation of SOS1cat-coupled HTL-MNPs towards the inner membrane in the tip of a neurite extension (4). Note that endogenous H-RAS is anchored at the inner face of the plasma membrane, cycling between its inactive GDP-bound and signaling active GTP-bound conformations (thick red lines).

### Generation of expression vectors and in-cell binding of HT fusion proteins to TMR-HTL

**Figure 2:**
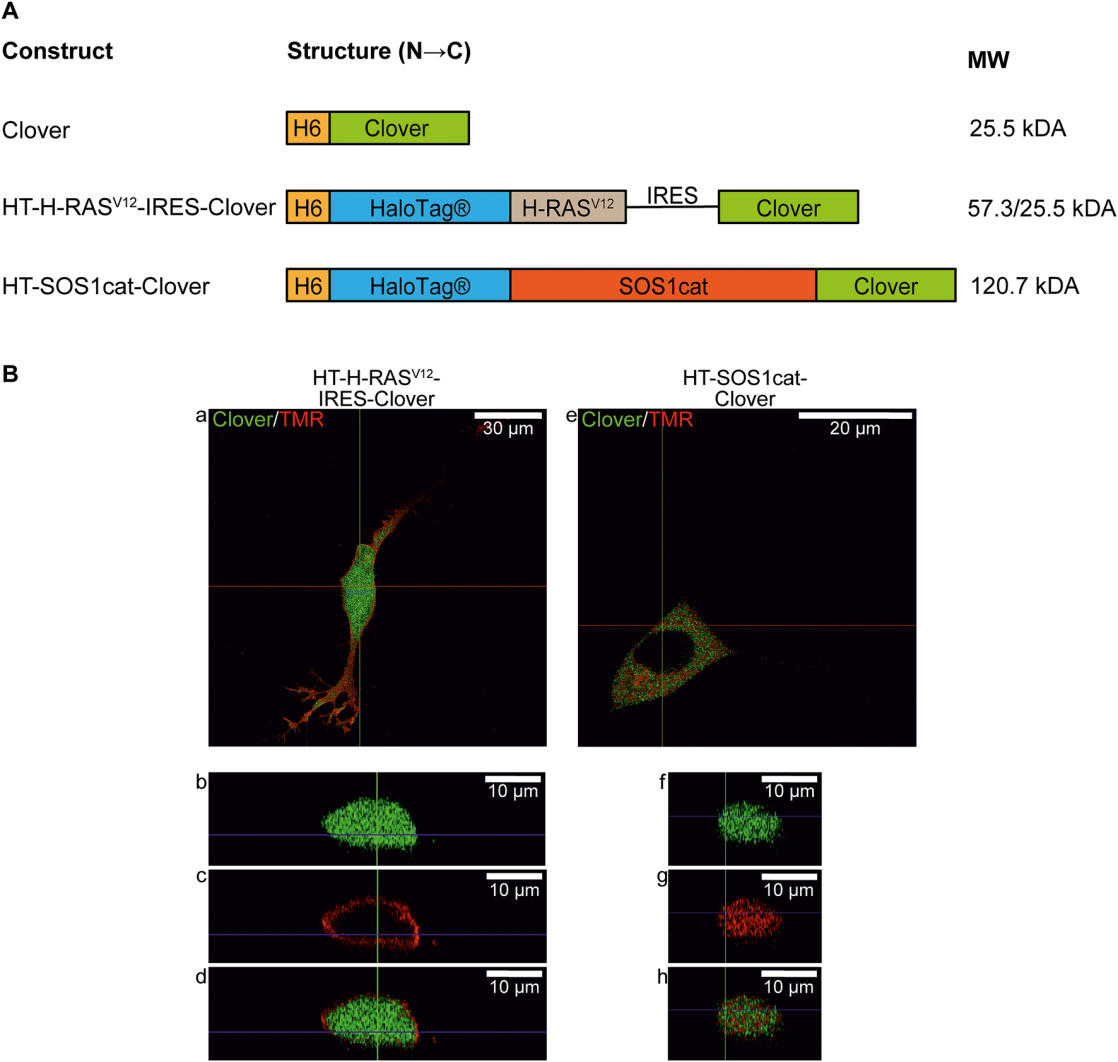
Domain structures of the constructs and intracellular distribution of expressed recombinant fusion proteins. (**A**) Constructs of the proteins. *H6 *poly(6)histidine tag, *H-RAS*^*V12*^ Harvey-RAS^V12^, *IRES *Internal Ribosome Entry Site, *SOS1cat *catalytic domain of son of sevenless 1. (**B**) Confocal imaging of SH-SY5Y cells transfected with either HT-H-RAS^V12^-IRES-Clover (a–d) or HT-SOS1cat-Clover (e–h) after in-cell binding of the red-fluorescent cell-permeable dye TMR-HTL 2 days after the transfection. Overlay images derived from green and red fluorescence channels are shown (a,e), and cross-sections indicated by the horizontal red line confirm that Clover is distributed equally in the cytoplasm and nucleus of HT-H-RAS^V12^-IRES-Clover-transfected cells (b,d), whereas HT-H-RAS^V12^ is exclusively located at the plasma membrane (c,d). In cells transfected with HT-SOS1cat-Clover, the Clover and TMR fluorescence are colocalized in the cytoplasm (f–h). For HT-SOS1cat-Clover, exclusion from the nucleus is visible (e).

To enable the magnetic translocation of fusion proteins within the cell, constitutively active H-RAS^V12^ or SOS1cat were bound to HTL-functionalized MNPs (Table 1). By using In-Fusion cloning technology, different fusion proteins were cloned into the vector backbone pTriEx-4 Neo (Fig. 2A). The sequence of the different constructs was confirmed by DNA sequencing. We transfected the cells to investigate the intracellular localization of the HT fusion proteins. After labeling with the membrane-permeable red fluorescent dye TMR-HTL, the cells were analyzed by confocal microscopy (Figs. 1B, 2B) and widefield microscopy (Supplementary Fig. 1). In transfected control cells expressing Clover only and in HT-H-RAS^V12^-IRES-Clover transfected cells, green fluorescence was visible all over the cells, because Clover was able to freely diffuse in the cytoplasm and to enter the nucleus through the nuclear pore complexes (Fig. 2B(a,b,d) and supplementary Fig. 1A(d,f) with corresponding line profiles in Supplementary Fig. 1B(j,l). In the case of HT-H-RAS^V12^-IRES-Clover transfected cells, the TMR ligand was mainly located at the plasma membrane, as predicted from the presence of the membrane anchoring CAAX (C = Cys, A = aliphatic, and X = any amino acid) box in the HT-H-RAS^V12^ fusion protein (Figs. 1B, 2B(c) and Supplementary Fig. 1A(i) with corresponding line profiles in Supplementary Fig. 1B(l). The selective membrane localization of HT-H-RAS^V12^ suggests its proper prenylation and palmitoylation.

**Table 1:** Results of MALS to characterize the in vitro binding of HT fusion proteins to HTL-MNPs.

Type	Condition	Mean r_h_ (nm)	SD (nm)	N
MNPs without HTL	Control	33.87	± 0.654	187
	BSAf	33.99	± 0.972	193
	HT-H-RAS^V12^	34.52	± 2.187	213
	HT-SOS1cat-Clover	33.79	± 0.637	212
MNPs with HTL	Control	40.31	± 1.451	135
	BSAf	40.65	± 2.644	82
	HT-H-RAS^V12^	42.52	± 1.239****	129
	HT-SOS1cat-Clover	44.89	± 3.054****	108

For MNPs without HTL, the addition of neither BSAf nor HT fusion proteins changed r_h_ significantly. Due to the presence of PEGylation and HTL, MNPs with HTL had an increased r_h_ compared to MNPs without HTL. Co-incubation with HT fusion proteins resulted in a significant increase of r_h_ that was proportional to the size of the proteins. In contrast, no significant change was detected for MNPs co-incubated with BSAf. ****P ≤ 0.0001, one-way ANOVA.

In contrast, the transfection of HT-SOS1cat-Clover resulted in a specific Clover signal in the cytoplasm and exclusion of fluorescence in the nucleus (Fig. 2B(e) and Supplementary Fig. 1A(e) with corresponding line profiles in Supplementary Fig. 1B(k). This nuclear exclusion was confirmed by TMR-HTL labeling (Fig. 2B(e) and Supplementary Fig. 1A(h) with corresponding line profiles in Supplementary Fig. 1B(k). The cross-section images of an exemplary xz-plane indicated by the horizontal red line in Fig. 2B(a,e) were corroborated by the results of the widefield microscopy shown in Supplementary Fig. 1.

### In vitro binding of HT fusion proteins to MNPs

To analyze the in vitro capturing of HT fusion proteins by MNPs, MNPs were measured by multiangle light scattering (MALS) after the addition of purified HT-H-RAS^V12^ and HT-SOS1cat fusion proteins (Table 1), as published recently^26^. The addition of HT-H-RAS^V12^ to HTL-MNPs significantly increased the hydrodynamic radius (r_h_) from 40.3 ± 1.5 nm to 42.5 ± 1.3 nm. For HTL-MNPs with HT-SOS1cat-Clover, r_h_ increased to 44.9 ± 3.1 nm. These changes in r_h_ are compatible with the difference in molecular weight (MW) of HT-H-RAS^V12^ (57.3 kDa) and HT-SOS1cat-Clover (120.7 kDa). The addition of fluorescein-labeled bovine serum albumin (BSAf) or HT fusion proteins to MNPs without HTL did not increase the r_h_ of the particles. Similar r_h_ values were observed with the addition of BSAf to HTL-MNPs, altogether showing that unspecific binding of BSA was not detectable (Table 1).

### Morphological changes in response to HT-H-RAS^V12^ and HT-SOS1cat-clover transfection

**Figure 3:**
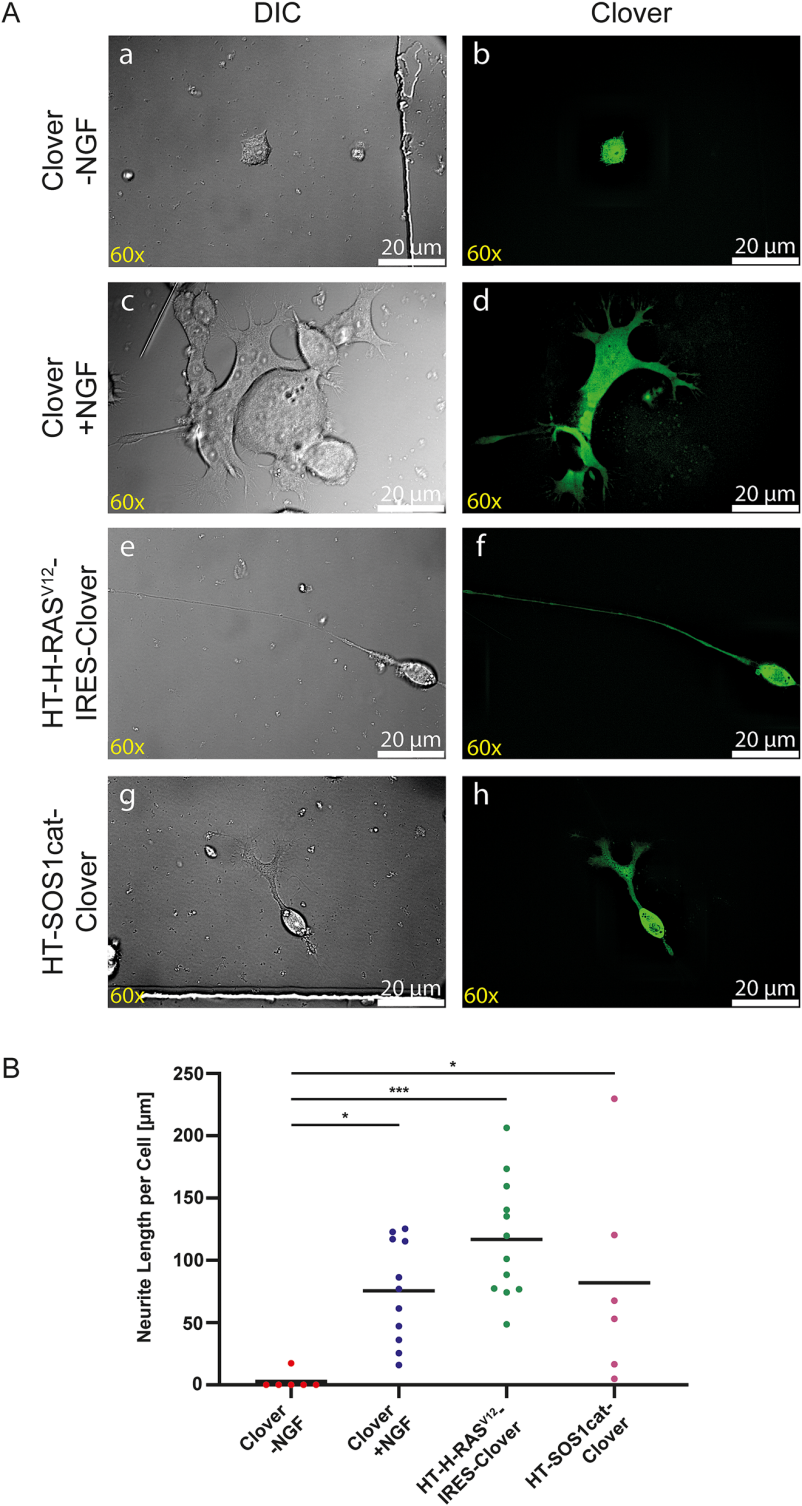
Morphological changes of PC12 cells upon the transfection of HT fusion proteins. (**A**) Wide-field (a,c,e,g) and fluorescent images (b,d,f,h). As a negative control, cells were only transfected with Clover and cultured in the absence of NGF (a,b). No morphological changes were induced, and the negative control cells appeared comparable to non-transfected cells when cultured in the absence of NGF (not shown). As a positive control, 50 ng/ml NGF was added to Clover-transfected cells for 2 days. An increase in cell volume, multinucleation, and outgrowth of cellular protrusions was detected in these cells (c,d). The transfection of PC12 cells with HT-H-RAS^V12^-IRES-Clover (e,f) or HT-SOS1cat-Clover (g,h) resulted in the outgrowth of neurites of different lengths. Scale bars correspond to 20 µm. (**B**) Comparison of neurite lengths. PC12 cells were transfected with Clover (control), HT-H-RAS^V12^-IRES-Clover, or HT-SOS1cat-Clover and cultivated for one day in proliferation medium followed by two days in differentiation medium. Clover-transfected PC12 cells were either cultured in the absence of NGF (− NGF; negative control) or with 50 ng/ml NGF (+ NGF; positive control). HT-H-RAS^V12^-IRES-Clover and HT-SOS1cat-Clover were cultured without NGF. (Clover − NGF: n = 6; Clover + NGF: n = 11; HT-H-RAS^V12^-IRES-Clover: n = 12; HT-SOS1cat-Clover: n = 6; two independent experiments; one-way ANOVA with Dunnett’s multiple comparisons test; confidence interval = 95%, *P ≤ 0.05; ***P ≤ 0.001).

PC12 cells respond to stimulation of the tropomyosin receptor kinase A (TRKA) receptor by NGF with phenotypical changes such as neurite extension and electrical excitability^27^. It was previously shown that constitutively active H-RAS^V12^ mimics these NGF effects if overexpressed in PC12 cells^28^ or after its cytoplasmic uptake of the purified protein in primary neurons^10^. We tested if HT fusion proteins remained functional after their expression in transfected PC12 cells (Fig. 3). As a control, cells were transfected with Clover and cultured with or without NGF. In the absence of NGF (negative control), Clover-transfected PC12 cells showed only minor morphological changes (average fiber length, 7.3 µm per cell; Fig. 3A(a,b),B). Incubation in differentiation medium containing 50 ng/ml NGF for 2 days (positive control) resulted in drastic morphological changes, such as the extension of cellular processes by all cells, with an average fiber length of 75.4 µm per cell (Fig. 3B), increased cell volume, and multinucleated cells (Fig. 3A(c,d)). Transfection with HT-H-RAS^V12^ resulted in an average fiber length of 116.8 µm per cell and transfection with HT-SOS1cat-Clover resulted in an average fiber length of 82.0 µm per cell after three days of culture (Fig. 3A(e–h),B).

To investigate the spatial distribution of HT-H-RAS in cells after neuronal differentiation, HT-H-RAS^V12^-IRES-Clover transfected PC12 cells were incubated for two days and labeled by TMR-HTL (see Fig. 1B). The three-dimensional reconstruction confirmed the membrane localization of H-RAS^V12^ as well as the cytoplasmic localization of Clover (Fig. 1B).

Taken together, both HT-H-RAS^V12^ and HT-SOS1cat-Clover were biologically active and capable of stimulating neurite induction and elongation in PC12 cells upon transfection.

### Magnetic manipulation of MNP-bound HT-SOS1cat-clover

**Figure 4:**
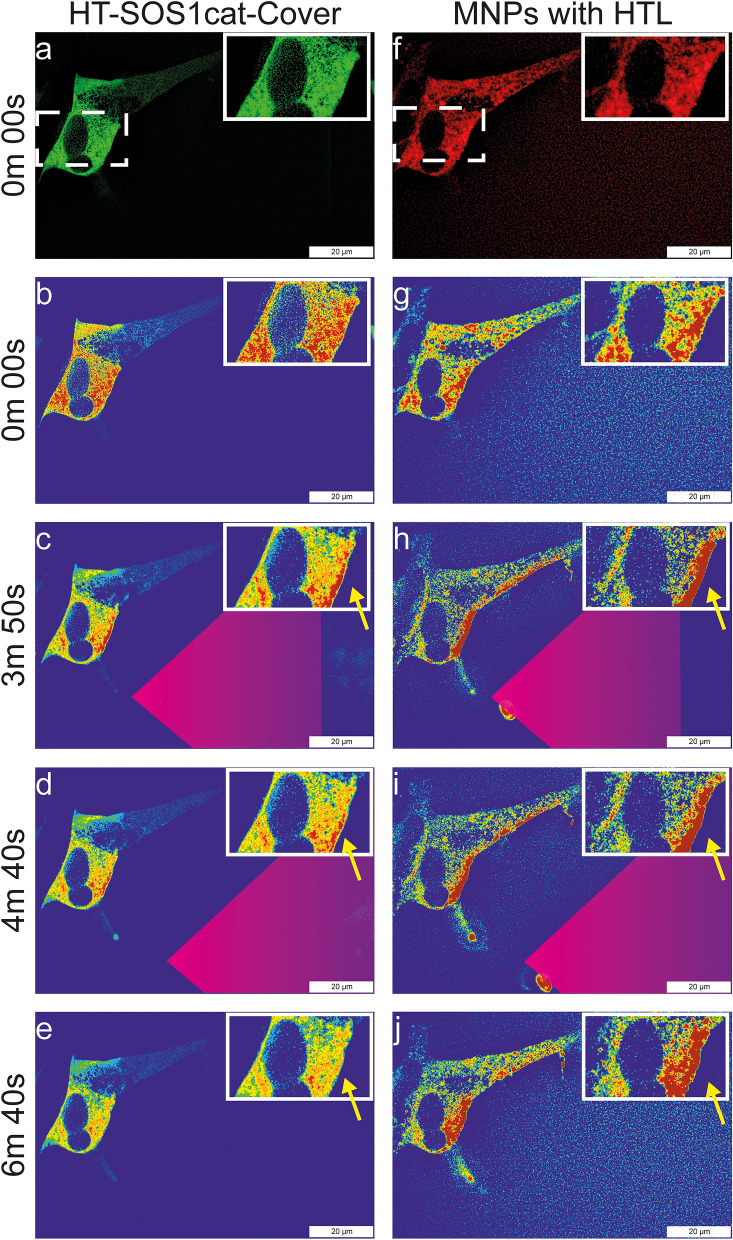
Magnetic manipulation of HT-SOS1cat-Clover-transfected SH-SY5Y cells upon the injection of HTL-MNPs. The fluorescence of HT-SOS1cat-Clover is shown in the left column (Supplementary Video S5), and the fluorescence of the HTL-MNPs is shown in the right column (Supplementary Video S6). For better visualization of the corresponding fluorescence intensities, a rainbow color scheme was applied, with red corresponding to high, yellow to medium, and blue to low fluorescence intensities (for HT-SOS1cat-Clover: Supplementary Video S7; for HTL-MNPs: Supplementary Video S8). The insets show magnifications of the ROI. (**a**,**b**) At 0 min 00 s, HT-SOS1cat-Clover was distributed homogeneously in the cytoplasm. (**f**,**g**) HTL-MNPs were slightly accumulated on the left and right sides of the nucleus of the cell in the ROI. (**h**) At 3 min 50 s, HTL-MNPs were attracted towards the magnetic tip and accumulated at the proximal plasma membrane. (**c**) Similar behavior was seen for HT-SOS1cat-Clover, although the change in fluorescence intensity was weaker. (**i**,**d**) When the tip was moved at 4 min 40 s, HTL-MNPs moved into the small neurite and an increase of HT-SOS1cat-Clover fluorescence was observed. (**j**,**e**) After the tip was removed, HTL-MNPs and HT-SOS1cat-Clover began to diffuse in the next two minutes. Scale bars correspond to 20 µm.

As a proof-of-principle for the in-cell capturing and magnetic translocation of protein-functionalized MNPs, we focused on HT-SOS1cat-Clover. We decided to perform these experiments in SH-SY5Y cells because the ratio of the cytoplasm to nucleus is higher than in PC12 cells, which allows for better live-cell imaging observation during the magnetic manipulation. Figure 4 shows the magnetic manipulation of HTL-MNPs (Fig. 4f, red fluorescence) in the cytoplasm of SH-SY5Y cells expressing HT-SOS1cat-Clover (Fig. 4a, green fluorescence). At 0.00 min, HTL-MNPs were distributed randomly in the investigated cells (Fig. 4g). In the cell shown in the inset, some MNP accumulation on the right and left cytoplasmic regions beside the nucleus was visible before the magnetic manipulation. For HT-SOS1cat-Clover, no asymmetric membrane accumulation was observed in the two cells that expressed the fusion protein (Fig. 4b). At 3 min 50 s, the magnetic tip (indicated by the purple triangle) was placed next to the cells, and a strong attraction of HTL-MNPs towards the magnetic tip was generated according to the local field gradient (for estimation of the force-distance relationship see Ref.^29^) (Fig. 4h). A comparable yet weaker response was found for HT-SOS1cat-Clover (Fig. 4c). The increased fluorescence intensity in the proximal membrane region was accompanied by decreased fluorescence intensity in the rest of the cell and could only be detected in the HT-SOS1cat-Clover-expressing cells. By moving the magnetic tip slightly towards the bottom of the image (4 min 40 s), HTL-MNPs and HT-SOS1cat-Clover fluorescence intensities increased even in the small neurite seen at the bottom-right side (Fig. 4d,i). After removing the magnetic tip, the HTL-MNPs and HT-SOS1cat-Clover partially diffused back from the membrane to the cytoplasm until 6 min 40 s (Fig. 4e,j; see also Supplementary Videos 1–8).

As a control, we performed the same experiment using MNPs lacking the HTL (Supplementary Fig. 2). Although the MNPs were clearly attracted towards the magnetic tip, the Clover fluorescence was unchanged in the cell cytoplasm. This observation demonstrates that within the cell, unspecific binding of fusion proteins to the MNPs was below the level of detectability.

Taken together, during magnetic translocation, the fluorescence intensities of MNPs (red) and of HT-SOS1cat-Clover (green) were co-localized exclusively with MNPs that carried covalently attached HTL. Thus, we obtained a differential tool to capture intracellular RAS-signal regulating proteins.

**Figure 5:**
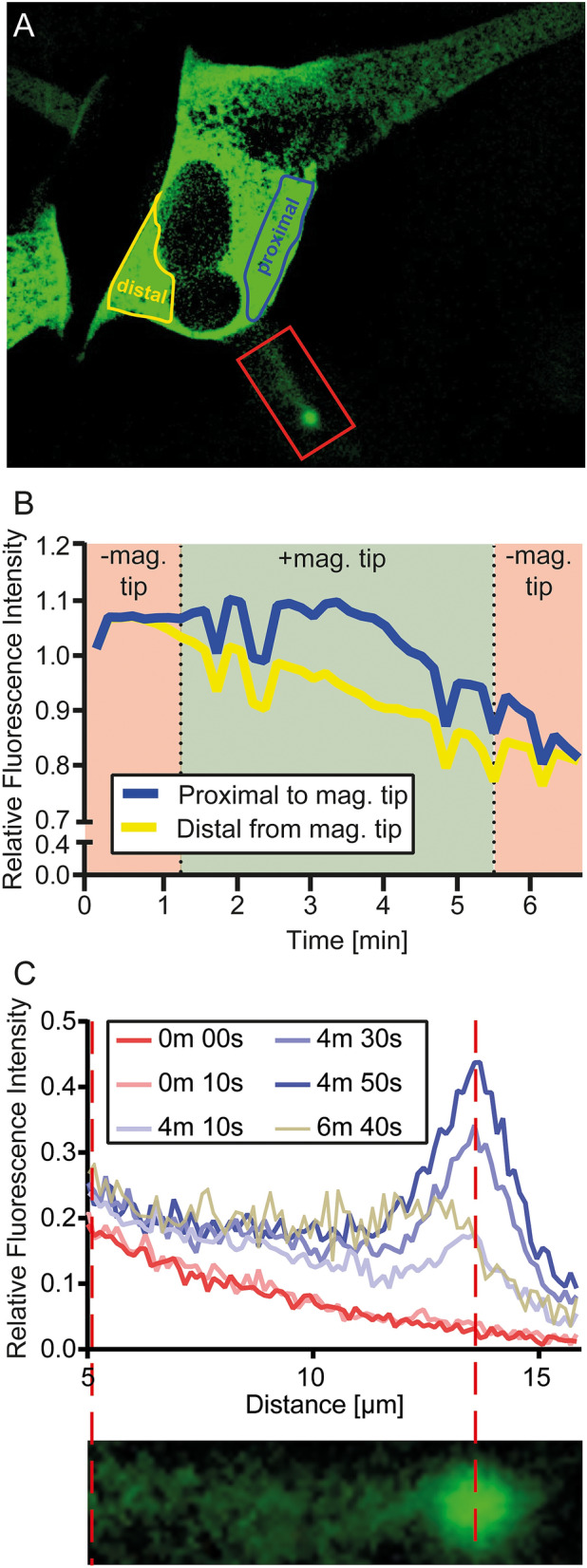
Analysis of the magnetic manipulation of HTL-MNP-bound HT-SOS1cat-Clover in SH-SY5Y cells. (**A**) An image of a SH-SY5Y cell expressing HT-SOS1cat-Clover injected with HTL-MNPs (MNP fluorescence not shown). The ROI of the cell body distal from the magnetic tip is indicated in yellow. The ROI of the cell body proximal to the magnetic tip is indicated in the region enclosed by the blue line. (**B**) Normalized and bleaching corrected RFI of both ROIs over time. − mag. tip indicates the period from when the magnetic tip was not in the field of observation (before 1 min 10 s and after 5 min 30 s). + mag. tip indicates when the magnetic tip was in the field of observation (1 min 10 s to 5 min 30 s). The differential area of fluorescence intensities during the application of the magnetic tip (+ mag. tip) between the blue (proximal to tip) and yellow (distal to tip) curves indicates a formal redistribution of HT-SOS1cat-Clover by about 12%. (**C**) The line profile of the fiber including its tip [red ROI in (**A**)] at different time points. The red broken lines indicate the position of the maximal fluorescence in the fiber´s tip.

In order to investigate in more detail the effects of the magnetic manipulation on MNPs and proteins, we compared the relative HT-SOS1cat-Clover-fluorescence intensity (RFI) of a region of interest (ROI) that is distal from the magnetic tip (Fig. 5A, encircled in yellow) with a ROI that is proximal to the magnetic tip (Fig. 5A, encircled in blue). Figure 5B shows the normalized and the bleaching-corrected RFI of the two ROIs over time. At the beginning of the experiment, no magnetic tip was applied, and the RFI in both ROIs were comparable. After about 40 s, the magnetic tip was moved towards the cell and reached the field of observation at 1 min 10 s (indicated by the left vertical dotted line). Although the magnetic tip was in a remote position, the magnetic attraction of the MNP was already observable and resulted in a minor loss of RFI in the yellow ROI, indicating that some HT-SOS1cat-Clover-coupled HTL-MNPs had translocated from the distal to the proximal ROI. When the magnetic tip was in proximity to the cell, the RFI in the yellow ROI was further decreased and accompanied by an increase of the RFI in the blue ROI. Because the membrane served as a barrier, HTL-MNP-bound HT-SOS1cat-Clover accumulated, resulting in an increased RFI due to the magnetic field induced redistribution (Fig. 5B) After the magnetic tip was removed (-mag. tip, Fig. 5B), the RFI in both ROIs reached comparable values due to the free diffusion of the HTL-MNPs in the cell body. By changing the position of the tip, HTL-MNPs were further guided to the neurite of the cell and finally accumulated in the neurite tip (Fig. 5A, enclosed in red).

The fluorescence intensities in the neurite were measured by analyzing the length of the neurite at different time points (Fig. 5C). Before the magnetic tip was applied, the RFI decreased with distance from the cell body (0 min 0 s, 0 min 10 s). When the magnetic tip was applied, HTL-MNP-bound HT-SOS1cat-Clover started to accumulate in the tip of the neurite. Although this event occurred as soon as the magnetic tip was applied, the accumulation was found to be enhanced later during the magnetic manipulation (Fig. 5C, see line profiles for 4 min 10 s, 4 min 30 s, 4 min 50 s). Upon removal of the magnetic tip at 5 min 30 s, the RFI decreased but not completely to the original values. In summary, MNPs functionalized with SOS1cat-Clover were successfully translocated by magnetic manipulation to the fiber’s tip.

### Upscaling cellular MNP loading and responsiveness to NGF

Towards the possible upscaling of cytoplasmic cell loading, we applied the scrape-loading method and analyzed to what extent the MNPs can be introduced into PC12 cells^30,31^. One day after the scrape-loading of MNPs, the cells were treated with or without 100 ng/ml NGF for two days. There was no significant difference in the loading efficiencies (Table 2). Notably, MNPs were found within about one third of all fibers (Table 2). After incubation of the scrape-loaded PC12 cells in differentiation medium containing 100 ng/ml NGF, at least one neurite was observed on 38.9 ± 13.1% of the cells, with an average length of 41.8 ± 11.2 µm (five independent experiments; 98 neurites in total were analyzed). This response to NGF by the neurite length was comparable to that observed in PC12 cell cultures without scrape-loading (Fig. 3B).

**Table 2:** Cellular MNP loading and responsiveness to NGF in PC12 cells.

	0 ng/ml NGF	100 ng/ml NGF
Loading efficiency of MNPs	41.5 ± 14.1%	63.9 ± 11.1%
Cells with at least one fiber	0.0	38.9 ± 13.1%
Average fiber length	na	41.8 ± 11.2 µm
Number of neurites per cell—mean	na	2.0 ± 0.5
Fibers with MNPs	na	34.4 ± 18.0%

One day after scrape-loading, cells were kept in proliferation medium without NGF (control) or in differentiation medium containing 100 ng/ml NGF for two days followed by analysis. Numbers are given as means ± SEM of four (without NGF; 378 cells were counted) or five (with NGF; 238 cells were counted) independent experiments (*na *not applicable).

## Discussion

In the nervous system, axon regeneration can be stimulated by activating intracellular signaling pathways such as H-RAS. The RAS/RAF/MEK/ERK signaling pathway is shown not only to play a major role in neuronal differentiation, migration, cytoskeletal dynamics, synaptic connectivity, neural protection, and apoptosis but also in the cytoskeletal reorganization and growth of axons (for review see Refs.^20,32^). Although these effects are promoted by the global neuronal activation of H-RAS, the stimulated neurite growth develops no intrinsic directionality in vitro or in vivo^20^. In order to achieve spatiotemporal control of the H-RAS-signaling pathway which is activated by the guanine nucleotide exchange factor SOS1cat, we report here the generation of fusion proteins that are able to bind to MNPs in vitro and in the cellular cytoplasm.

We confirmed the HT-H-RAS^V12^ was localized at the membrane, while HT-SOS1cat-Clover was localized in the cytoplasm of SH-SY5Y cells. We were able to induce fiber outgrowth in PC12 cells by the overexpression of these fusion proteins. Furthermore, we demonstrated the intracellular capturing of HT-SOS1cat-Clover after the injection of HTL-MNPs. Finally, using a magnetic tip, we demonstrated the controlled redistribution of MNPs within the cell including a pronounced accumulation of HTL-MNP-bound SOS1cat-Clover in the tip of neurite extensions.

The precise control of cellular events and functions are of key relevance for regenerative medicine^33^. Previously, the magnetic control of lamellipodia formation by MNP-bound T-lymphoma invasion and metastasis-inducing protein 1 (TIAM) and GEFs of RHO-GTPases was reported^34^. Because the RAS/RAF/MEK/ERK pathway is crucial for fiber outgrowth in CNS neurons^20^, we chose a constitutively active H-RAS^V12^ and the catalytic domain of its GEF, SOS1cat, to control neurite growth. We used the HT system to bind the recombinant proteins to chemically synthesized MNPs. The HT is a modified haloalkane dehalogenase that can covalently bind to chloroalkanes^35^.

We generated HT fusion proteins of H-RAS^V12^ and SOS1cat for cellular expression (Fig. 2). To monitor the cytoplasmic mobility of the fusion protein when bound to HTL-MNPs, we cloned a Clover-tagged version of SOS1cat fusion proteins and determined its GEF activity^26^. In order to preserve the C-terminal membrane anchoring mechanism of HT-H-RAS^V12^, we chose to generate an internal ribosome entry site (IRES) construct that expressed HT-H-RAS^V12^ and Clover as separate proteins to detect transfected cells (Fig. 2).

As a first step, we used the MALS technique to test the in vitro binding of proteins to MNPs^26,36^. We showed a significant size-dependent increase in r_h_ upon the addition of HT-H-RAS^V12^ or HT-SOS1cat-Clover to HTL-MNPs (Table 1).

Subsequently, we tested the subcellular localization of the HT fusion proteins by taking advantage of the membrane-penetrable fluorescent HTL-TMR ligand (Figs. 1B, 2B, and Supplementary Fig. 1), which can bind covalently to HT. SH-SY5Y cells transfected with Clover and HT-H-RAS^V12^-IRES-Clover showed Clover fluorescence in the cytoplasm and the nucleus. Clover is a monomeric GFP variant with a size of 27 kDa and freely diffuses through the nuclear pore complex, which has a cut-off of approximately 60 kDa^37^. In contrast, HT-SOS1cat-Clover is localized in the cytoplasm and excluded from the nucleus because of its size of 120.7 kDa. Although HT-SOS1cat-Clover is an artificial construct, its subcellular localization is comparable to full-length SOS1 in the absence of a signaling stimulus. With respect to the binding of HTL-TMR, unspecific binding was not detected in Clover-transfected cells in the absence of the HT. For HT-SOS1cat-Clover, the colocalization of green and red fluorescence was detected in living cells by both fluorescence and confocal microscopy, and for HT-H-RAS^V12^-IRES-Clover, the membrane localization of HT-H-RAS^V12^ was clearly visible with both types of microscopy. These results suggest farnesylation and palmitoylation occurred for the plasma membrane anchoring of HT-H-RAS^V12^^38^.

The biological activity of the HT fusion proteins with respect to neurite initiation and growth was tested in PC12 cells. PC12 cells are derived from rat pheochromocytoma tumor cells and respond to NGF under serum-free conditions with differentiation to sympathetic neuron-like cells including features such as electrical excitability, the expression of neuronal markers, and neurite extensions^27^. It was shown previously that this effect can be mimicked by H-RAS^V12^ overexpression^28^. We assessed neurite induction and growth upon the transfection of the HT fusion protein constructs. The transfection of Clover alone did not trigger the induction of neurite growth, but the addition of NGF resulted in multinucleated cells with increased cell volume and neurites. Consistent with previously published data^28^, transfection with HT-SOS1cat-Clover and HT-H-RAS^V12^-IRES-Clover both resulted in significant neurite growth (Fig. 3B).

Next, we demonstrated the remote-control location of subcellular HT-SOS1cat-Clover-MNPs by magnetic forces. We chose HT-SOS1cat-Clover for the proof-of-principle experiment since we confirmed its cytoplasmic localization and biological activity as a fusion protein. MNPs with or without HTL were successfully microinjected into the cytoplasm of the target cells, where they distributed uniformly (Fig. 4 and Supplementary Fig. 2). In addition, both types of MNPs responded instantly to the magnetic field gradient of the magnetic tip, and spatial control was possible by moving the tip. We were able to achieve the asymmetrical alignment of HT-SOS1cat-Clover-bound HTL-MNPs along the cytoplasmic sub-membrane regions and the accumulation of HT-SOS1cat-Clover-bound HTL-MNPs in the neurite extensions (Fig. 5). The attraction of HTL-MNP-bound HT-SOS1cat-Clover resulted in an increased RFI of up to 12% in the ROI that was close to the magnetic tip (Fig. 5B). In contrast to the free cytoplasmic mobility in the cell body regions (Fig. 5B), the redistribution of HTL-MNPs after removing the magnetic field was somehow restricted in the neurites (Fig. 5C). In order to respond to external stimuli, neurites have highly active cytoskeletal dynamics^39^ within the small volume of the neurite. Previous experiments in dorsal root ganglion (DRG) neurons revealed that the cytoskeletal density in neurites is dependent on a neurite-inducing stimulus^40^. We, therefore, hypothesized that the denser meshwork of the cytoskeleton in neurites might impede the free diffusion of MNPs in the neurites. This enhanced retention time of MNPs might help contribute to a persistent growth response even after the magnetic field is removed.

Recent advances in stem cell replacement therapies allow the production of a large number of progenitor cells^41,42^. A prerequisite for the magnetic guidance of neurite or axonal tips in cell replacement therapy in the brain is the generation of a method that provides a large population of MNP-loaded neurons. Previously, it was shown that H-RAS^V12^ activates protein kinase C within minutes after scrape-loading^43^. We found that the scrape-loading of cells with MNPs is possible and that MNP-loaded cells are fully responsive to NGF within two days (Table 2). These observations further suggest no obvious toxic effect on NGF-induced fiber length by the scrape-loading of MNPs and that long-time (days) magnetic treatments by devices such as coaxially arranged Halbach cylinders can be tested in the future^44,45^.

In conclusion, directed axonal growth of CNS neurons is required in regenerative medicine to establish physiological reinnervation upon brain injury or disease. CNS neurons encounter a growth-inhibiting environment and thus their capability of regeneration is restricted^6^. Experiments of growth cone guidance due to magnetic forces after MNPs are taken up into the cell into endosome-like structures have been described^22,23^, but this approach has not been confirmed for guiding the MNPs over the long distances of the nervous system. We present here a complementary approach targeting the neuroprotective and neurite-inducing RAS/RAF/MEK/ERK pathway based on protein-functionalized MNPs within the cytoplasm^26^. We demonstrate the biological activity of HT-H-RAS^V12^ and HT-SOS1cat fusion proteins and show their binding to MNPs in vitro and in vivo.

As transplantation of induced pluripotent stem cell-derived dopaminergic neurons in vivo is already established^42,46–48^, we consider our results as an initial step to advance tools for refining cell replacement therapies based on neurons loaded with functionalized magnetic nanoparticles targeting neurite growth signaling in Parkinson model systems^20,26^.

## Methods

A detailed list of the used chemicals, kits, and devices is provided in the supplements.

### Cell culture

#### Culturing of SH-SY5Y and PC12 cells

SH-SY5Y is a subclone of the human neuroblastoma cell line SK-N-SH^49^. SH-SY5Y cells were a kind gift from Franz-Josef Klinz (University of Cologne, Germany). The cells were cultured in a 1:1 mixture of Roswell Park Memorial Institute medium (RPMI) and Dulbecco’s Modified Eagle’s Medium high glucose (DMEM), supplemented with 10% fetal bovine serum, 1% penicillin/streptomycin, and 1% glutamine in a T75 flask at 37 °C and 5% CO_2_.

PC12 cells are a pheochromocytoma cell line of the rat adrenal medulla and were a kind gift from Stefan Wiese (Ruhr-Universität Bochum, Germany). The cells were cultured in poly-d-lysine-coated (PDL) T75 flasks in DMEM supplemented with 10% horse serum (HS), 5% FBS, and 1% P/S (proliferation medium) in a T75 flask at 37 °C and 5% CO_2_.

For differentiation, the proliferation medium was replaced with DMEM supplemented with 1% HS, 1% FBS, 1% P/S (differentiation medium). Depending on the experiment, 0, 50, or 100 ng/ml nerve growth factor (NGF) was added. Further details are described in the supplementary information.

### Synthesis and HaloTag ligand functionalization of γ-Fe_2_O_3_@SiO_2_ core–shell nanoparticles (HTL-MNPs)

The synthesis of γ-Fe_2_O_3_@SiO_2_ core–shell nanoparticles and their functionalization with HTL as well as PEG4 was performed as recently published^26^. A summary of the procedure is attached as a supplement.

### Generation and analysis of HT-H-RAS^V12^ and HT-SOS1cat fusion proteins

#### Cloning of HT constructs

All constructs used were cloned into the pTriEx-4 Neo vector. This vector allows the expression in *E. coli*, vertebrate, and insect cell lines.

The HT-TEV sequence was taken from the pFN18 HT T7 Flexi Vector. The sequence of the green fluorescent protein variant Clover was a kind gift from Simon Ebbinghaus (TU Braunschweig, Germany). Plasmids with the sequence of human H-RAS^V12^ (accession number NP_005334.1) and human SOS1cat (accession number AAA35913) were a kind gift from Alfred Wittinghofer (MPI Dortmund, Germany). In the case of constitutively active H-RAS^V12^, the amino acid at position 12 was changed from glycine to valine. To generate the SOS1cat fusion protein, the amino acids 550–1050 of SOS1, which include the RAS exchanger motif (REM) and the cell division cycle 25 (cdc25) domain, were used^50^.

All constructs were generated with In-Fusion HD Cloning Plus according to the manufacturer's manual. The In-Fusion primers (Supplementary Table S1) were designed with the corresponding online tool (https://www.takarabio.com/learning-centers/cloning/in-fusion-cloning-tools) and SnapGene (GSL Biotech LLC, Chicago, IL, United States) plugin. All constructs were confirmed by an in-house DNA sequencing service.

### Analysis of morphological effects of HT-H-RAS^V12^ and HT-SOS1cat-clover in PC12 cells

For the analysis of morphological alterations upon HT-H-RAS^V12^ or HT-SOS1cat-Clover expression, PC12 cells were electroporated with H-RAS^V12^-IRES-Clover, SOS1cat-Clover, or Clover (control) by using the Amaxa Cell Line Nucleofector Kit V (Lonza). 5 × 10^4^ electroporated PC12 cells were seeded on PDL-coated µ-dishes with a grid. After 24 h, the proliferation medium was removed, and differentiation medium with or without NGF was added. The cells were analyzed after 2 days in differentiation medium by using fluorescence microscopy.

### Binding analysis of HT fusion proteins

#### Multiangle light scattering (MALS) for characterization of in vitro biofunctionalized MNPs

To analyze the binding of HT fusion proteins to HTL-functionalized MNPs, we used the MALS technique as previously published^26^. In brief, MNPs were sonicated (40 kHz, 480 W) for 4 min at room temperature and afterward centrifuged at 3800×*g*. Recombinant expressed HT fusion proteins were mixed with MNPs from the supernatant, incubated at room temperature for 3 min, and kept on ice until they were analyzed.

The flow cell of the miniDAWN TREOS II (Wyatt Technology) was washed with ddH_2_O and further with 1 × PBS until a noise-free forward scattering and stable signal was achieved. The samples were injected using a 1 ml syringe in fractions of approximately 200 µl.

### In-cell binding assay of HT fusion proteins

To analyze the functional binding of the HT fusion proteins to a ligand, SH-SY5Y cells were electroporated with plasmids coding for HT-H-RAS^V12^-IRES-Clover, HT-SOS1cat-Clover, or Clover (Control) by using the Amaxa Cell Line Nucleofector Kit V. 4 × 10^4^ electroporated cells per well were seeded on PDL-coated 8-well µ-slides. After 48 h of incubation at 37 °C and 5% CO_2_, the cells were labeled with the membrane-permeable fluorescent dye tetramethylrhodamine (TMR)-HTL according to the manufacturer’s manual. Labeled cells were further analyzed by fluorescence and confocal microscopy.

### Magnetic manipulation of MNPs

To test the magnetic manipulation of MNPs inside living cells, we chose SH-SY5Y cells, because their higher cytoplasmatic volume allows for better microscopic observation compared to PC12 cells. 4 × 10^4^ SH-SY5Y cells were seeded on 35 mm culture dishes. After 24 h incubation at 37 °C and 5% CO_2,_ the cells were sufficiently attached for microinjection.

MNPs were sonicated (40 kHz, 480 W) for 4 min at room temperature and afterward centrifuged at 3800×*g*. 3 µl of the supernatant was transferred into a microcapillary (Femtotip). The solution was then injected via FemtoJet 4i (Eppendorf) into the cytoplasm of the desired cell.

For the magnetic manipulation, a self-made magnetic tip was used as previously published^29^. A 200 µl pipette tip was glued to the screwable part of a Femtotip. The pipette tip was shortened by approximately 3 mm, and a 5 × 1.5 × 1 mm N45 Neodymium magnet was inserted to about half its length. To focus the magnetic field, a 6–8 mm long piano string with a diameter of 0.4 mm was attached to one side of the magnet. A longer piece of piano string was pulled over a flame until it tore into two parts that both had parabolic ends. Afterward, the string was shortened to the desired length.

The three-dimensional control of the magnetic tip was facilitated by InjectMan 4 (Eppendorf).

### Scrape-loading of PC12 cells with MNPs

Scrape-loading of PC12 cells was performed essentially as described previously^51^. PC12 cells were seeded at a density of 2 × 10^5^ cells per well in PDL-coated 35 mm dishes in proliferation medium. The next day, the medium was removed, and the cells were washed once with PBS. MNPs were diluted to a final concentration of 300 µM in PBS supplemented with 1 mg/ml bovine serum albumin (BSA), and 100 µl of this MNP-BSA-PBS solution was applied to the cells per dish. The rubber lip of a cell scraper was scratched several times over the entire adherent cells. Cells were collected in 1 ml proliferation medium and transferred into a 1.5 ml reaction tube. After centrifugation for 4 min at 150×*g* and room temperature in a 1.5 ml reaction tube, the supernatant was removed, and the pellet was resuspended in 1 ml proliferation medium. The cells were seeded on PDL-coated µ-dishes with a grid. The next day, the medium was exchanged for differentiation medium containing 100 ng/ml recombinant human β-NGF. In the case of scrape-loaded control cells, proliferation medium without NGF was added. The cellular localization of the MNPs was analyzed by fluorescence microscopy with the same fluorescence intensity and exposure time after two days.

### Fluorescence microscopy

Cell imaging, microinjection, and magnetic manipulations were performed with an Olympus IX83 microscope, and the images were acquired with a digital camera (Hamamatsu Orca Flash4.0 V3). A 60 × water immersion objective (Olympus) was used. Non-fluorescent images were acquired in differential interference contrast (DIC) mode. For fluorescent images, single bandpass filters were used.

### Processing and analysis of fluorescent images

Basic processing and analysis of fluorescent images were performed using an Olympus cellSense Dimension (version 2.1, Olympus Soft Imaging Solutions GmbH, Münster, Germany, https://www.olympus-lifescience.com/en/software/cellsens/). Further analysis was performed with Fiji (version 1.52p, https://imagej.net/Fiji)^52^ and GraphPad Prism (version 8.1.1 for Microsoft Windows, GraphPad Software, San Diego, California, United States, www.graphpad.com).

To analyze the asymmetric distribution of HTL-MNP-bound HT-SOS1cat-Clover, first, bleaching correction was performed. The intensities of equally distributed HT-SOS1cat-Clover in SH-SY5Y cells in several regions of interest (ROIs) were measured over time. The average Clover fluorescence intensities were plotted, normalized, and fitted to a single-exponential function. Fitting was used to determining the parameters characterizing the fluorescence decay by bleaching, with *Y*_*0*_ being the y-axis intersection, *Plateau* the value at infinite times, and *k* the exponential rate constant. After that, intensity data were corrected using the determined parameters and Eq. (1):

1 $${Y}_{corr}={Y}_{raw}/(\left({Y}_{0}-Plateau\right)\times {e}^{\left(-k\times X\right)}+Plateau).$$

For SH-SY5Y cells containing HTL-MNP-bound HT-SOS1cat-Clover, one ROI at the side of the cell that was close to the magnetic tip, and one ROI that was distant from the magnetic tip was chosen. The average Clover fluorescence intensities of the ROIs were normalized and bleaching corrected using Eq. (1).

### Confocal microscopy

Confocal images were acquired with an TCS SP8 confocal microscope (Leica Microsystems GmbH, Wetzlar, Germany) and 63 × water immersion objective. Live cell imaging was performed in an incubation chamber at 37 °C and 5% CO_2_. Each image was taken with a 3 × line average. The Z-stack distance was adjusted using the “optimized mode”. The images were processed and analyzed with LAS X software (version 3.5, Leica Microsystems GmbH, Germany, https://www.leica-microsystems.com/products/microscope-software/p/leica-las-x-ls/).

## Supplementary Information

Supplementary Video 1.

Supplementary Video 2.

Supplementary Video 3.

Supplementary Video 4.

Supplementary Video 5.

Supplementary Video 6.

Supplementary Video 7.

Supplementary Video 8.

Supplementary Information.

## Data Availability

The datasets used and/or analyzed during the current study are available from the corresponding author upon request.
