# Supplementary material for: Magnetic spatiotemporal control of SOS1 coupled nanoparticles for guided neurite growth in dopaminergic single cells
Source: Sci Rep. 2020 Dec 31;10:22452. doi: 10.1038/s41598-020-80253-w (PMC7775457; doi:10.1038/s41598-020-80253-w)
Supplement: Supplementary file 9 — Supplementary Information. [file 41598_2020_80253_MOESM9_ESM.pdf]

Scientific Reports

Magnetic spatiotemporal control of SOS1 coupled nanoparticles for guided neurite growth in dopaminergic single cells

Fabian Raudzus [1,†], Hendrik Schöneborn [2], Sebastian Neumann [3], Emilie Secret [4], Aude Michel [5], Jérome Fresnais [6], Oliver Brylski [7], Christine Ménager [8], Jean-Michel Siaugue [9] and Rolf Heumann [10,*]

[1]Ruhr-Universität Bochum, Faculty of Chemistry and Biochemistry, Department of Biochemistry II – Molecular Neurobiochemistry, 44801 Bochum, Germany; fabian.raudzus@ruhr-uni-bochum.de; ORCID: 0000-0001-8327-2076

[†]Present address: Kyoto University, Center for iPS Cell Research and Application (CiRA), Department of Clinical Application, 606-8507 Kyoto, Japan; fabian.raudzus@cira.kyoto-u.ac.jp

[2]Ruhr-Universität Bochum, Faculty of Chemistry and Biochemistry, Department of Biochemistry II – Molecular Neurobiochemistry, 44801 Bochum, Germany; hendrik.schoeneborn@ruhr-uni-bochum.de; ORCID: 0000-0002-8913-2987

[3]Ruhr-Universität Bochum, Faculty of Chemistry and Biochemistry, Department of Biochemistry II – Molecular Neurobiochemistry, 44801 Bochum, Germany; sebastian.neumann@ruhr-uni-bochum.de; ORCID: 0000-0003-3706-9012

[4]Sorbonne Université, CNRS, Physico-chimie des Électrolytes et Nanosystèmes Interfaciaux, PHENIX, F-75005 Paris, France; emilie.secret@sorbonne-universite.fr; ORCID: 0000-0003-2200-7265

[5]Sorbonne Université, CNRS, Physico-chimie des Électrolytes et Nanosystèmes Interfaciaux, PHENIX, F-75005 Paris, France; aude.michel@upmc.fr

[6]Sorbonne Université, CNRS, Physico-chimie des Électrolytes et Nanosystèmes Interfaciaux, PHENIX, F-75005 Paris, France; jerome.fresnais@sorbonne-universite.fr; ORCID: 0000-0002-2037-0637

[7]Technische Universität Braunschweig, Institut für Physikalische und Theoretische Physik, Biophotonik, Rebenring 56, 38106 Braunschweig, Germany; o.brylski@tu-braunschweig.de

[8]Sorbonne Université, CNRS, Physico-chimie des Électrolytes et Nanosystèmes Interfaciaux, PHENIX, F-75005 Paris, France; christine.menager@sorbonne-universite.fr; ORCID: 0000-0002-4080-3508

[9]Sorbonne Université, CNRS, Physico-chimie des Électrolytes et Nanosystèmes Interfaciaux, PHENIX, F-75005 Paris, France; jean-michel.siaugue@sorbonne-universite.fr; ORCID: 0000-0003-1217-9493

[10]Ruhr-Universität Bochum, Faculty of Chemistry and Biochemistry, Department of Biochemistry II – Molecular Neurobiochemistry, 44801 Bochum, Germany; rolf.heumann@ruhr-uni-bochum.de; ORCID: 0000-0003-4364-2655

*****Correspondence: rolf.heumann@ ruhr-uni-bochum.de; Tel.: +49 234 32-28230

Supplementary Information

Supplementary Figure legends

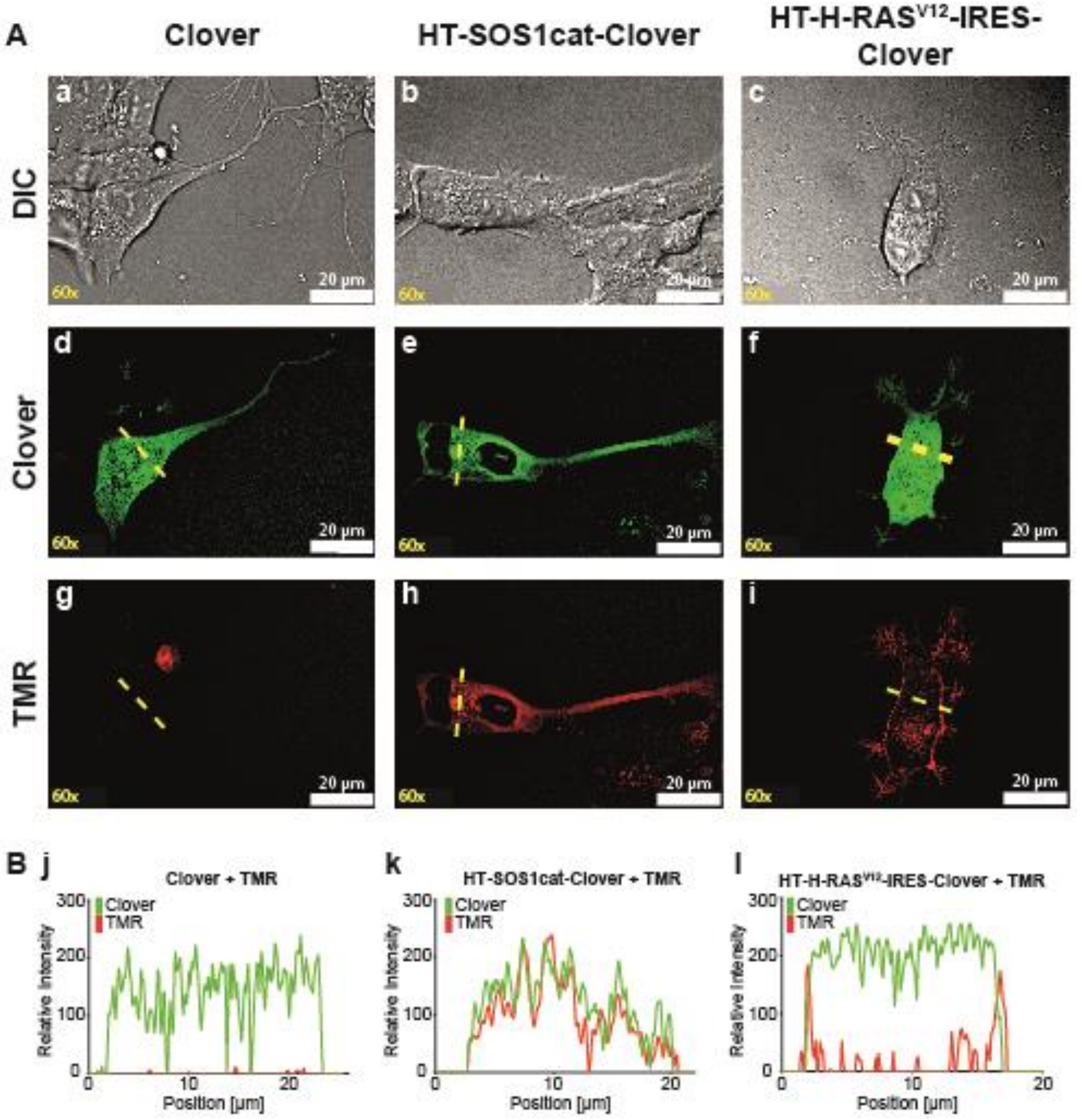

Supplementary Figure 1. Fluorescence imaging of the in-cell binding of TMR-HTL to HT fusion proteins. (A) SH-SY5Y cells were transfected with either Clover (a, d, g), HT-SOS1cat-Clover (b, e, h), or HT-H-RAS^{V12}-IRES-Clover (c, f, i). Images a-c were obtained in the differential interference contrast (DIC) mode; images d-f show the fluorescence in the Clover channel (green); and images g-i show the fluorescence in the TMR channel (red). In the case of Clover (d) and HT-H-RAS^{V12}-IRES-Clover (f), green fluorescence was located in the cytoplasm and nucleus. In contrast, HT-SOS1cat-Clover (e) only showed fluorescence in the cytoplasm. No specific TMR signal was detectable for Clover-transfected cells (g). For HT-SOS1cat-Clover-transfected cells (h), the TMR signal was colocalized with the Clover fluorescence. The fluorescence of the TMR ligand

was mainly present at the plasma membrane in the case of HT-H-RAS^{V12}-IRES-Clover-transfected cells (i). Some occasional spotty fluorescence was seen in nuclear regions, but this fluorescence was not further analyzed because of its absence in the confocal images (Fig. 2B). (B) The intensities of Clover and TMR fluorescence for each condition are shown as line profiles. The relative intensities of both channels are plotted for each pixel across the yellow broken lines in (A). The green line illustrates the Clover fluorescence, and the red line illustrates the TMR fluorescence. Scale bars correspond to 20 µm.

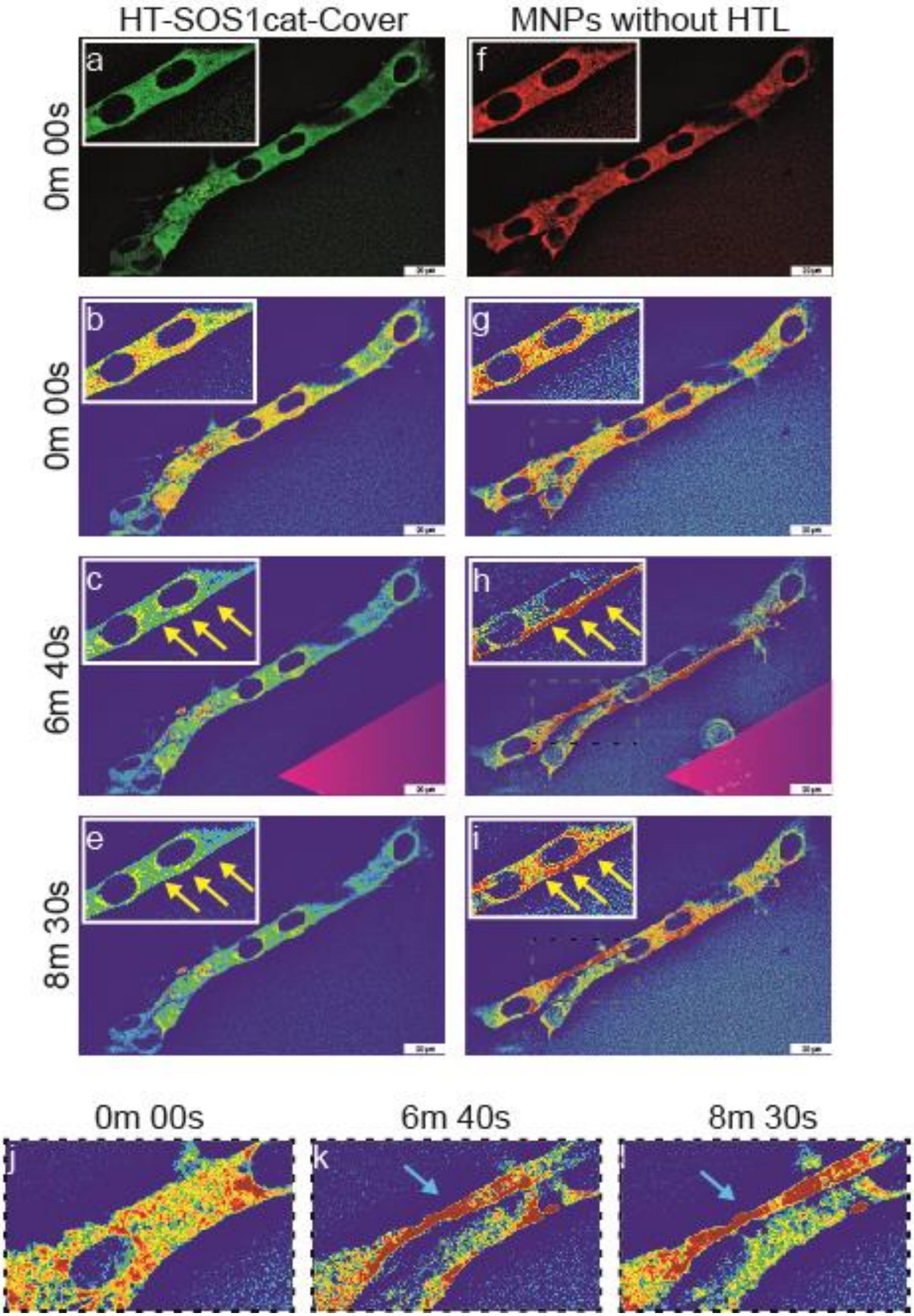
HT-SOS1cat-Cover
MNPs without HTL
0m 00s
0m 00s
6m 40s
8m 30s
a
b
c
e
f
g
h
i
0m 00s
6m 40s
8m 30s
j
k
l

Supplementary Figure 2. Magnetic manipulation of HT-SOS1cat-Clover-transfected SH-SY5Y cells upon the injection of MNPs without HTL excludes the covalent binding of HTL-fusion proteins. The fluorescence of HT-SOS1cat-Clover is shown in the left column (Supplementary Video S1), and the fluorescence of the MNPs is shown in the right column (Supplementary Video S2). For clearer visualization of the corresponding fluorescence intensities, a rainbow color scheme was applied, with red corresponding to high, yellow to medium, and blue to low fluorescence intensities (for HT-SOS1cat-Clover: Supplementary Video S2; for MNPs: Supplementary Video S4). The insets show magnifications of a region of interest (ROI). (b) At 0 min 00 sec, HT-SOS1cat-Clover was distributed homogeneously in the cytoplasm. (g) MNPs were slightly accumulated around the nuclei. (h) When the magnetic tip (indicated by the magenta-colored shape) was brought into close proximity of the cells, the strong attraction of MNPs towards the tip was detectable by an increase in the fluorescence intensity at the proximal plasma membrane (position of highest accumulation indicated by yellow arrows). (c) Conversely, no change in the HT-SOS1cat-Clover distribution was visible. (i) After the tip was removed, most of the MNPs diffused back into the cytoplasm. Interestingly, the MNPs stayed in their "manipulated position" in the neurite (i, magnified in j-l and indicated by the broken black line, indicated by the turquoise arrows). (e) The fluorescence intensity of HT-SOS1cat-Clover was unchanged after the magnetic tip was removed. (j-l) Magnification of MNPs in the neurite at three different time points. The scale bars correspond to 20 µm.

Supplementary Video legends

Supplementary Video 1. Magnetic manipulation of HT-SOS1cat-Clover-transfected SH-SY5Y cells upon the injection of MNPs without HTL – Clover Fluorescence.

Supplementary Video 2. Magnetic manipulation of HT-SOS1cat-Clover-transfected SH-SY5Y cells upon the injection of MNPs without HTL – rainbow color scheme of Clover fluorescence.

Supplementary Video 3. Magnetic manipulation of HT-SOS1cat-Clover-transfected SH-SY5Y cells upon the injection of MNPs without HTL – rhodamine fluorescence.

Supplementary Video 4. Magnetic manipulation of HT-SOS1cat-Clover-transfected SH-SY5Y cells upon the injection of MNPs without HTL – rainbow color scheme of rhodamine fluorescence.

Supplementary Video 5. Magnetic manipulation of HT-SOS1cat-Clover-transfected SH-SY5Y cells upon the injection of MNPs with HTL – Clover fluorescence.

Supplementary Video 6. Magnetic manipulation of HT-SOS1cat-Clover-transfected SH-SY5Y cells upon the injection of MNPs with HTL – rainbow color scheme of Clover fluorescence.

Supplementary Video 7. Magnetic manipulation of HT-SOS1cat-Clover-transfected SH-SY5Y cells upon the injection of MNPs with HTL – rhodamine fluorescence.

Supplementary Video 8. Magnetic manipulation of HT-SOS1cat-Clover-transfected SH-SY5Y cells upon the injection of MNPs with HTL – rainbow color scheme of rhodamine fluorescence.

List of used materials and devices

µ-dishes with grid, 81166, ibidi GmbH, Martinsried, Germany
35 mm dish, Nunc, 153066, Thermo Fisher Scientific, Waltham, MA, USA
60x water immersion objective, Olympus, Shinjuku, Tokyo, Japan
8-well µ-slides, 80826, ibidi GmbH, Martinsried, Germany
Amaxa Cell Line Nucleofector Kit V, Lonza Group Ltd., Basel, Switzerland
APTS, Sigma-Aldrich, St. Louis, Missouri, United States
cell scraper, 83.1830, Sarstedt, Newton, NC, USA
DMEM, D6171, Sigma-Aldrich, St. Louis, Missouri, United States
FBS, Biochrom GmbH, Berlin, Germany
FemtoJet 4i, Eppendorf, Hamburg, Germany
Femtotip, 5242952008, Eppendorf, Hamburg, Germany
Glutamine, Sigma-Aldrich, St. Louis, Missouri, United States
Hamamatsu Orca Flash4.0 V3, Hamamatsu City, Japan
HS, Sigma-Aldrich, St. Louis, Missouri, United States
In-Fusion HD Cloning Plus, Takara Bio Inc., Kusatsu, Shiga, Japan
InjectMan 4 (Eppendorf, Hamburg, Germany)
Leica SP8, Leica Microsystems GmbH, Wetzlar, Germany
miniDAWN TREOS II, Wyatt Technology, Santa Barbara, CA, United States
N45 Neodymium magnet, Magnet-Shop.net, Lohr am Main, Germany
Olympus IX83 microscope, Olympus, Shinjuku, Tokyo, Japan
P/S, Sigma-Aldrich, St. Louis, Missouri, United States
PDL, P6407, Sigma-Aldrich, St. Louis, Missouri, United States
PEOS, ABCR, Karlsruhe, Germany
pFN18 HaloTag T7 Flexi Vector, G2751, Promega, Fitchburg, WI, United States
piano string, 20820-20950, Pianelli, Jena, Germany
pTriEx-4 Neo vector, 70933, Merck KGaA, Darmstadt, Germany
Recombinant Human β-NGF, 450-01, Peprotech, Hamburg, Germany
RPMI-1640, R7388, Sigma-Aldrich, St. Louis, Missouri, United States
single bandpass filters, Chroma Technology Corp., Bellows Falls, Vermont, United States
TMR-HTL, G8251, Promega, Fitchburg, WI, United States

Supplementary Methods

Construct Name	Original Template	Primer name	Sequence (5'-3')
HT-H-RAS^{V12}	HaloTag	HT_FOR	*ATCAAAGGAGATATACCATGCATCATCACCAT*
		HT_REV	*ATTCTGTCGGGTTATCGCTCTGAAAGTACAGATCCTCAG*
	H-RAS^{V12}	HT-RAS_FOR	*GAGCGATAACCCGACAGAATACAAGCTTGTTGTTGT*
		HT-RAS_REV	*GTGTATACAGCTGTGCGGCCTCAGGAGAGCACACACTTGCAG*
HT-H-RAS^{V12}-IRES-Clover	HaloTag	HT_FOR	*ATCAAAGGAGATATACCATGCATCATCACCAT*
		HT_REV	*ATTCTGTCGGGTTATCGCTCTGAAAGTACAGATCCTCAG*
	H-RAS^{V12}	HT-RAS-Clover_FOR	*GAGCGATAACCCGACAGAATACAAGCTTGTTGTTGT*
		HT-RAS-Clover_REV	*TCGAATTCCAGGAGAGCACACACTTGC*
	pDream-Clover-Ruby2	Clover-RAS_FOR	*TGTGCTCTCCTGGAATTCGATGGTGTCAAAGG*
		Clover-RAS_REV	*GTGTATACAGCTGTGCGGCCGGCGGCGGTGAC*
	pIRES2-HTN-Nurr1-dtTomato	IRES_FOR	*GCAAGTGTGTGCTCTCCTAACCCCTCTCCCTCCCCCCC*
		IRES_REV	*CACCACTCCAGTAAACAGTTCCTCGCCCTTGCTCACCA*
HT-SOS1cat	HaloTag	HT_FOR	*ATCAAAGGAGATATACCATGCATCATCACCAT*
		HT_REV	*TCCTTTCCAGGTTATCGCTCTGAAAGTACAGATCCTCAG*
	SOS1cat	SOS1cat_FOR	*GAGCGATAACCTGGAAAGGATGCTTGATGTAACAATGC*
		SOS1cat_REV	*GTGTATACAGCTGTGCGGCCCATGGTACCTGGTCTTGGGTTTGATG*
HT-SOS1cat-Clover	HaloTag	HT_FOR	*ATCAAAGGAGATATACCATGCATCATCACCAT*
		HT_REV	*TCCTTTCCAGGTTATCGCTCTGAAAGTACAGATCCTCAG*
	SOS1cat	SOS1cat-Clover_FOR	*GAGCGATAACCTGGAAAGGATGCTTGATGTAACAATGC*
		SOS1cat-Clover_REV	*TCGAATTCCACATGGTACCTGGTCTTGGGT*
	pDream-Clover-Ruby2	Clover-SOS1cat_FOR	*AGGTACCATGTGGAATTCGATGGTGTCAAAGGGG*
		Clover-SOS1cat_REV	*GTGTATACAGCTGTGCGGCCGGCGGCGGTGAC*

Supplementary Table S1. List of primers used

Cell Culture

SH-SY5Y cells were passaged at least once per week or when the cell monolayer reached approximately 80% confluence. PC12 cells were passaged once per week. Cells were washed twice with 1x phosphate-buffered saline (PBS) and detached with 5 ml trypsin (2.5 mg/ml)/ethylenediaminetetraacetic acid (EDTA) (0.02 mol/l) in 1x PBS. As soon as most of the cells started to detach, the trypsin was inhibited by 5 ml complete medium. The cells were transferred into a 15 ml centrifugation tube and centrifuged at 200 x *g* for 3 min. The supernatant was discarded, and the pellet was resuspended in 1 ml fresh complete medium. For further

culturing, the desired number of cells was transferred in a new T75 flask with 10 ml complete medium. For further experiments, the cells were counted using a Neubauer chamber, and the necessary cell number was seeded on a new culture vessel.

Synthesis and HaloTag Ligand Functionalization of γ-Fe_2O_3@SiO_2 Core-Shell Nanoparticles (HTL-MNPs)

The synthesis of magnetic core-shell nanoparticles begins with the synthesis of γ-Fe_2O_3 cores by an inverse co-precipitation of iron salts in an ammonia solution. After oxidation of the obtained Fe_3O_4 magnetite nanoparticles, the maghemite nanoparticles are size-sorted in order to have less polydisperse population. These γ-Fe_2O_3 cores are then used in a Stöber silica condensation process to encapsulate them in a silica shell. The silica shell is made fluorescent by the addition of silane-functionalized rhodamine B, and the colloidal stability of the synthesized nanoparticles is ensured by their functionalization with aminopropyltriethoxysilane (APTS) and silanized-poly(ethylene glycol) short chains (3–[methoxy(polyethyleneoxy)propyl]trimethoxysilane, PEOS) [1].
The amine group on the surface of the synthesized nanoparticles also allows for their further functionalization with a HaloTag ligand (HTL), which can bind specifically to HT-fused proteins. The functionalization with HTL is done in a two-step process [2]. The MNPs were first functionalized with dibenzocyclooctyne-PEG4-N-hydroxysuccinimidyl ester. Then the dibenzocyclooctyne moiety reacted through click chemistry with the azide moiety on the azido-functionalized HTL. Finally, the remaining amine groups at the surface of the MNPs were converted to carboxylic acid groups through a reaction with succinic anhydride in order to have negatively charged particles. Details on the synthesis and functionalization of the MNPs were published recently [3].

References

1 Georgelin, T., Bombard, S., Siaugue, J.-M. & Cabuil, V. Nanoparticle-Mediated Delivery of Bleomycin. *Angewandte Chemie International Edition* **49**, 8897-8901, doi:10.1002/anie.201003316 (2010).

2 Etoc, F. *et al.* Magnetogenetic Control of Protein Gradients Inside Living Cells with High Spatial and Temporal Resolution. *Nano Letters* **15**, 3487-3494, doi:10.1021/acs.nanolett.5b00851 (2015).

3 Schöneborn, H. *et al.* Novel Tools towards Magnetic Guidance of Neurite Growth: (I) Guidance of Magnetic Nanoparticles into Neurite Extensions of Induced Human Neurons and In Vitro Functionalization with RAS Regulating Proteins. *Journal of Functional Biomaterials* **10**, 32, doi:10.3390/jfb10030032 (2019).
